# Use of Pegvisomant in acromegaly. An Italian Society of Endocrinology guideline

**DOI:** 10.1007/s40618-014-0146-x

**Published:** 2014-09-23

**Authors:** A. Giustina, M. R. Ambrosio, P. Beck Peccoz, F. Bogazzi, S. Cannavo’, L. De Marinis, E. De Menis, S. Grottoli, R. Pivonello

**Affiliations:** 1 Struttura Ambulatoriale di Endocrinologia, A.O. Spedali Civili di Brescia, University of Brescia, Via Biseo 17, 25100 Brescia, Italy; 2 Department of Medical Science, University of Ferrara, Ferrara, Italy; 3 Endocrinology, Fondazione IRCCS Ca’ Granda Policlinico, University of Milano, Milan, Italy; 4 Section of Endocrinology, Department of Clinical and Experimental Medicine, University of Pisa, Pisa, Italy; 5 Department of Clinical and Experimental Medicine, University of Messina, Messina, Italy; 6 UOS Patologia Ipofisaria, Policlinico Universitario A. Gemelli, Rome, Italy; 7 Internal Medicine, Montebelluna Hospital, Montebelluna, Italy; 8 Endocrinology, Diabetology and Metabolism, AO Città della Salute e della Scienza di Torino, University of Torino, Turin, Italy; 9 Dipartimento di Medicina Clinica e Chirurgia, Sezione di Endocrinologia , Università Federico II, Naples, Italy

**Keywords:** Pegvisomant, Acromegaly, Somatostatin analogs, Growth hormone, IGF-1

## Introduction

Acromegaly management is a significant challenge for endocrinologists. The Acromegaly Consensus Group developed several statements on the management of acromegaly and specifically on its medical treatment [1–3]. Acromegaly is a quite rare condition generally caused by a growth hormone (GH)-secreting pituitary adenoma [4]. Delayed diagnosis leads to prevalent presentation of the disease at the stage of macroadenoma (two-thirds of patients) and frequent persistence of active disease after surgery which remains in many patients the primary treatment option [5]. However, active acromegaly is potentially a life threatening condition due its severe systemic complications [6, 7] Therefore, elevated GH and insulin-like growth factor (IGF)-1 levels need to be strictly controlled after failure of surgery with medical or radiation treatments [8]. Furthermore, criteria for disease control may not be fulfilled in a considerable proportion of patients undergoing medical treatment with somatostatin receptor ligands (SRLs) after unsuccessful surgery [9, 10]. Accordingly, some acromegaly patients require the administration of GH antagonist Pegvisomant [11]. Pegvisomant has been introduced in clinical practice more than a decade ago as a medical therapy of acromegaly. However, specific guidelines for Pegvisomant use in acromegaly are lacking. Therefore, the Italian Society of Endocrinology constituted a task force with the objective of assessing the published literature and the clinical experience with Pegvisomant. This group involved endocrinologists recognized experts in the field of acromegaly management and their understanding of the data reported so far worldwide as well as their recommendations for Pegvisomant use in clinical practice are presented here. Biochemical and clinical results of Pegvisomant, indications, treatment modalities, combination therapies, safety and regulatory and cost/efficacy issues were evaluated. Evidences were graded with GRADE system [1–3, 12, 13] based on the quality of evidence as very low quality (VLQ; expert opinion with one or a small number of small uncontrolled studies in support), low quality (LQ; large series of small uncontrolled studies), moderate quality (MQ; one or a small number of large uncontrolled studies or meta-analyses), or high quality (HQ; controlled studies or large series of large uncontrolled studies with sufficiently long follow-up). Recommendations were defined discretionary (DR) if based on VLQ-LQ evidence, or strong (SR) if supported by MQ-HQ evidence.

## What is Pegvisomant

Pegvisomant is a drug designed to block the GH receptor (GHR) and, therefore, GH action. The discovery of this GHR antagonist was made possible by the elucidation of the structure–function relationship of GH and its receptor [11, 14]. Growth hormone is a 22 kDa polypeptide with 191 amino acids, two disulphide bonds and four alpha helices synthesized in the anterior pituitary and central to regulation of growth and differentiation. It has many other biological actions including enhancement of protein synthesis, lipolysis and hyperglycemic effects. Although GH may have direct effects on peripheral tissues most of its growth promoting effects are mediated by IGF-1 [15–17]. Growth hormone has two distinct domains (sites one and two) that interact with preformed GHR dimer on plasma membrane triggering conformational changes required for signaling [18]. The affinity of GH binding site one for GHR is high whereas the affinity of site two is lower. After initial high affinity binding at site one, subsequent binding at site two produces functional receptor dimerization. After the GH/GHR interaction, a series of intracellular signaling systems is mobilized, resulting in the activation or inactivation of genes responsible for GH action [19].

Pegvisomant is a GH analog with a single-aminoacid substitution at position 120 that generates the antagonist. Additional changes include amino acid substitutions within binding site 1 and a further modification by the addition of polyethylene glycol moieties [20]. The GHR antagonist acts by failing to induce proper or functional GHR dimerization. The pegylated [polyethylene glycol (PEG)] counterpart Pegvisomant is generated by the conjugation of GHR antagonist with four or five moieties of PEG 5000; PEG molecule addition increases the size of the antagonist and its serum half life from ~30 min to more than 100 h, by reducing renal clearance and intravascular proteolysis, and reduces immunogenicity of the molecule [21]. Like GH, the GHR antagonist has a relatively small size (22 kDa), and is normally cleared via the kidneys and/or GHR internalization [22].

## Biochemical outcomes in trials and observational registries

Circulating GH values are not useful as biochemical marker of Pegvisomant effects in acromegaly both because endogenous GH secretion may increase during treatment due to negative feedback and, particularly, due to cross-reactivity of GH with Pegvisomant in most GH assays [21] (HQ). Therefore, GH should not be measured in monitoring Pegvisomant treatment (SR). Normalization of IGF-1 levels represents the main end point of Pegvisomant treatment (HQ) [23, 24] although sudden and remarkable GH increase during Pegvisomant therapy could be a marker of tumor re-growth [25] (VLQ). Many studies reported IGF-1 normalization or marked reduction in acromegaly patients treated with Pegvisomant [26] (HQ). In addition, improvement in quality of life was suggested even adding Pegvisomant in patients already effectively controlled by SRLs [27] (VLQ). However, reported effectiveness of Pegvisomant varied widely depending on the type of study (clinical trial vs. observational) as it happens with other medical therapies in acromegaly [3] (MQ). Indeed, serum IGF-1 levels normalized in more than 90 % of patients particularly in initial clinical trials [28–32], while the control rate was lower in studies performed in the clinical setting and based on the retrospective analysis of disease-specific databases [33–39] (Table 1). Inadequate dose titration, poor compliance to daily injections, suboptimal selection of patients and technical problems related to IGF-1 assay could justify a lower than expected efficacy in “real life” conditions (VLQ), since the existence of a true “biochemical resistance” to Pegvisomant, as observed with SRLs [40], has not been clearly documented yet (VLQ). Effectiveness of Pegvisomant may be inversely correlated to baseline IGF-1 levels and starting dose should be higher and dose titration more rapid in patients with a worse endocrine profile (VLQ) [26, 41]. Better efficacy of Pegvisomant was associated with male gender, leanness, lower baseline GH and/or IGF-1 levels, previous irradiation, and related to treatment duration and appropriate dose titration (LQ) [37, 38, 41]. The role of d3GHR polymorphism, which could modify receptor sensitivity to GH [42], in response to Pegvisomant is still controversial (VLQ) [43–45]. Availability of validated assays is crucial for monitoring appropriately effectiveness of treatment and dose titration (SR). For this reason, IGF-1 values should be measured with the same method over time in each patient (SR). At present, considerable differences exist among available assays, due to lack of standardization, use of different types of antibodies and interference of binding proteins (MQ) [46]. Moreover, specific age-related normative intervals are rarely obtained, as recommended by available general guidelines [10], in local populations by centralized laboratories (LQ). Finally, given the within-individual biological variation of IGF-1 assays caution should be also used in interpreting values close to reference limits even if obtained with the same method [47, 48] (DR).

**Table 1:** Summary of biochemical results with Pegvisomant treatment in clinical trials and observational/retrospective studies in acromegaly

Author	Primary end point	N. of patients	Disease control (%)	Dose of Pegvisomant	Duration of the study
Randomized clinical trials:					
Herman-Bonert et al. [28]	IGF-1 normalization	3	100	30–80 mg/weekly	6 weeks
		3	100	10–20 mg/day	3 months
Trainer et al. [29]	Dose-related efficacy	109	10	placebo	33 months
			38	10 mg/day	3 months
			75	15 mg/day	3 months
			82	20 mg/day	3 months
van der Lely et al. [30]	IGF-1 normalization	90	97	–	12 months
		62	92	–	18 months
Drake et al. [31]	IGF-1 normalization	7	100	20 mg/day (median; range 15-40)	24 months
Barkan et al. [32]	IGF-1 normalization	49	78	16 mg/day (mean; range 5-40)	8 months
Colao et al. [26]	IGF-1 normalization	12	75	25 mg/day (median; range 10-40)	12 months
Observational or retrospective studies:					
Schreiber et al. [33]	IGF-1 normalization	147	64	16.5 mg/day (mean; range 10-50)	6 months
		102	71		12 months
		39	76		24 months
Higham et al. [34]	IGF-1 normalization	11	95	15 mg/day (median; range 10-60)	91 months
Trainer [35]	IGF-1 normalization	792	62	15 mg/day (median in controlled patients)	60 months
				16 mg/day (median in not controlled patients)	
Buchfelder et al. [36]	IGF-1 normalization	273	56	15 mg/day (median)	6 months
		202	71		24 months
		133	71		36 months
		71	65		48 months
		24	58		60 months
Marazuela et al. [37]	IGF-1 normalization	44	84	17 ± 7 mg/day in men 16 ± 8 mg/day in women	23 months (mean)
Garsia Basavilbaso et al. [38]	Duration-related efficacy	28	46	9.6 mg/day (mean)	3 months
			59		
					6 months
van der Lely et al. [39]	Safety and efficacy	1288	63	18 mg/day (mean in controlled patients) 20 mg/day (mean in uncontrolled patients)	43 months (mean)

## Peripheral and tissue effects of Pegvisomant

Treatment with Pegvisomant improves clinical syndrome of acromegaly in a high percentage of patients (HQ), positively impacts glucose metabolism (MQ), quality of life (MQ) and cardiovascular and skeletal complications (MQ) [49] (Table 2).

**Table 2:** Clinical and comorbidity outcomes of Pegvisomant therapy in acromegaly

Endpoints	Results	References
**Glucose metabolism**		
Fasting glucose levels		[52–54]
Glucose tolerance		[53, 58]
HbA1c %		[33, 53]
Insulin sensitivity		[52, 55–57]
HOMA index		[52, 55]
**Lipid metabolism**		
Total cholesterol		[59, 60] / [26, 61]
LDL cholesterol		[59, 60] / [26, 61]
Triglyceride		[59, 60] / [26, 61]
Lipoprotein (a)		[59, 60]
**Cardiovascular complications**		
Cardiac mass		[63]
Systolic and diastolic function		[63]
Rhythm disturbances		[64]
Blood pressure		[26, 61]
Framingham risk score		[61]
Carotid arteries wall thickness		[65]
Brachial arteries vascular function		[65]
**Skeletal complications**		
Bone turn-over		[71, 72]
BMD		[73]

* Denote significant change

### Glucose and lipid metabolism

In acromegaly, abnormal glucose tolerance, insulin resistance, hyperinsulinemia and diabetes mellitus are frequently observed [50] (HQ). Medical treatment of acromegaly may variably influence glucose metabolism. It is known that SRLs inhibit insulin secretion, inducing a possibly negative impact on glucose homeostasis (MQ) [51], whereas Pegvisomant improves insulin sensitivity likely by ameliorating IGF-1 excess and its effect on insulin resistance (MQ) [33, 52–57]. Several studies demonstrated that Pegvisomant monotherapy induced a significant decrease in fasting glucose levels and HbA1c [33, 52–54, 58] also in patients with diabetes mellitus and impaired glucose tolerance (MQ). A positive impact of Pegvisomant on peripheral insulin sensitivity was also demonstrated [52, 55–57] (MQ). However, a substantial proportion of patients included in these studies were resistant to SRLs; therefore, improved glucose metabolism could derive from better biochemical control and/or to removed inhibitory effect of SRLs on insulin secretion [58] (VLQ). Variable results were observed on lipid metabolism after Pegvisomant. An increase in total and LDL cholesterol with unchanged triglyceride levels and a significant decline in lipoprotein (a) levels was observed [59, 60], whereas other authors [24, 61] reported that lipid profile did not change during Pegvisomant therapy (LQ).

### Cardiovascular and skeletal complications

Acromegaly is associated with a specific cardiomyopathy, characterized by biventricular hypertrophy and complicated by initial diastolic dysfunction and late systolic dysfunction, potentially leading to heart failure (HQ) [62]. Furthermore, systemic arterial hypertension, frequently associated with the disease, contributes to worsening acromegalic cardiomyopathy [62]. Long-term (18 months) treatment with Pegvisomant induced a significant reduction of cardiac mass and significant improvement of diastolic and systolic function in patients with acromegaly mostly resistant to SRLs (LQ) [63]. Treatment with Pegvisomant could also exert beneficial effects on rhythm disorders and hyperkinetic syndrome (LQ) [64]. Moreover, 12 months of Pegvisomant therapy were associated with improved blood pressure, particularly of diastolic values, in hypertensive patients [24, 61] (LQ). IGF-I normalization significantly lowered predicted cardiovascular risk, calculated with the Framingham risk score [61] (LQ). On Pegvisomant slight reduction of carotid arteries wall thickness and significant improvement of brachial arteries vascular function in patients with acromegaly resistant to SRLs were reported (VLQ) [65].

Growth hormone and IGF-I play a significant role in the regulation of bone metabolism [66, 67] (HQ). Acromegaly increases risk of vertebral fractures not necessarily associated with reduced bone mass (MQ) [68–70] but with increased bone turn-over which normalized during 6 months of Pegvisomant treatment [71, 72] (LQ). Long-term treatment with Pegvisomant also induced a significant increase of bone mineral density in active acromegaly (LQ) [73]. Although Pegvisomant use was weakly associated with an increased rate of fractures this has been attributed to global increased severity of the disease in treated patients [70] (LQ).

## Indications

Pegvisomant is traditionally indicated for treatment of acromegaly patients with inadequate response to pituitary adenomectomy or radiation therapy, or for those intolerant or resistant to SRLs (HQ). However, a clear-cut definition of resistance to SRLs is missing (VLQ) [74]. In fact, during SRL therapy biochemical control is defined as random basal GH lower than 1 mcg/liter and IGF-1 levels below the upper limit of normal range for age (MQ) [3]. Using these strict criteria [10] normalization of biochemical activity in unselected patients with acromegaly after long-term (>6–12 months) treatment with maximal SRL doses occurs approximately in 25–50 % of cases [3, 75–77] (MQ). Non-responders to SRL therapy (minimal effect on GH and IGF-I levels and on tumor shrinkage) should be switched to Pegvisomant (SR). In partial responders to SRLs, Pegvisomant monotherapy or combination therapy with Pegvisomant and SRL should be considered (DR). Tumor shrinkage quite frequently (around 50 % of treated patients) occurs during therapy with SRLs often but not necessarily together with biochemical normalization [78–80] (MQ). Interestingly, in patients with acromegaly and McCune Albright syndrome surgery and even radiation therapy often can not be performed [81] and SRLs have very low chances to be effective [81] (LQ). In these patients, Pegvisomant can be considered as primary treatment (DR). Moreover, primary post-surgical medical treatment with Pegvisomant should be considered in patients already proven to be resistant to SRLs as those who underwent a sufficiently long (>3–6 months) trial of pre-surgical SRL treatment which demonstrated to be ineffective in controlling GH and IGF-1 (unless a > 75 % surgical debulking is achieved [82]) (DR). Primary post-surgical Pegvisomant treatment can be considered in patients after irradiation in whom elevated IGF-1 levels may persist for long time but likelihood of tumor regrowth is modest [1] (DR) and in patients with poorly controlled diabetes mellitus in whom SRLs may potentially worsen glucose metabolism [51–54] (DR).

## Treatment modalities

Pegvisomant is administered by subcutaneous injections. Ten, 15, and 20 mg per vial are available dosages. Initially, treatment regimens contemplated a 40–80 mg loading dose. In clinical practice this procedure has not proven to be useful and has been abandoned (LQ) [23]. Daily administration is the most effective because it achieves higher serum Pegvisomant concentrations with a lower dose of drug (MQ) [29, 83]. The target of therapy is to achieve serum IGF-I in the middle of age-related reference range (MQ) [11]. Starting dose is usually 10 mg/day and maximum maintenance dose which currently can be administered based on regulatory indications is 30 mg daily (LQ) [84]. For patients who require a dose >20 mg daily, Pegvisomant treatment is more inconvenient due to daily multiple injections (VLQ) [85]. After treatment start, serum IGF-I levels fall within 2 weeks and then reach a plateau after 4 weeks (HQ) [29]. Consequently, it is suggested to measure IGF-I 4 to 6 weeks after beginning treatment and after every change of dose until biochemical control is reached (DR). Once serum IGF-I levels are normalized, they should be monitored every 3–6 months [38] since Pegvisomant dose may require up- or down-titration in the same individual during treatment (DR) [3].

## Combination therapies

### Dopamine agonists

Cabergoline, a dopamine receptor agonist, has limited activity when used as monotherapy in acromegaly (MQ) [86, 87]. However, its combination with SRLs was shown to be effective in some patients (LQ) [86]. Few data are available regarding the combination of cabergoline and Pegvisomant. However, it was reported that addition of Pegvisomant to cabergoline as well as of cabergoline to Pegvisomant may result in improved IGF-1 control (LQ) [88, 89]. A better response was associated with baseline IGF-1 levels not higher than 160 % of ULN. No correlation was found with baseline prolactin levels. The combined treatment was well tolerated and safe (LQ).

### Somatostatin receptor ligands

When compared with monotherapy, combination treatment with SRLs may require a lower dose (even in only 1 weekly administration) of Pegvisomant to obtain similar efficacy (MQ) (Table 3) [90–93]. This is due to different mechanisms, including elevation of serum Pegvisomant levels [93], reduced insulin concentration in the portal vein, which decreases the number of available liver GH receptors [94] and reduced endogenous GH levels (LQ). In all reported trials, combination treatment was generally well tolerated (LQ). However, transient liver function test abnormalities were observed in a variable percentage of cases (11–38 %), apparently higher when compared with monotherapy. Significant tumor shrinkage during combined treatment was observed in 13–19 % of patients [95]. Glucose metabolism was not substantially affected [96].

**Table 3:** Studies investigating the efficacy and safety of adding PEG to SRLs in patients with uncontrolled acromegaly

	Design of the study	N. of patients	Length of the study, median (range)	Mean age (SD)	SRL treated patients (%)	IGF-1 at baseline, mean (SD)	PEG dose, median (range)	IGF-1 at EOS, mean (SD)	Patients with normal IGF-1 at EOS (%)	TLEE (%)
Feenstra et al. [90]	Prospective	19	42 weeks	51 years (12.6)	Lanreotide ATG 120 mg/4 weeks (81 %) Octreotide LAR 30 mg/4 weeks (19 %)	510 ng/ml (229)	60 mg weekly (40–80 mg)	187 (92)	95	38
Neggers et al. [91]	Prospective	32	138 weeks (35–149)	53 years (12.8)	Lanreotide ATG 120 mg/4 weeks (69 %) Octreotide LAR 30 mg/4 weeks (31 %)	428 ng/ml (220)	60 mg, weekly or biweekly (40–160)	137 ng/ml (47)	100	34
Van der Lely. [92]	Prospective	57	28 weeks	51.6 years (12.7)	Lanreotide ATG 120 mg/4 weeks (100 %)	NA	60 mg, weekly or biweekly (40–120)	NA	57.9	11
Jorgensen et al. [93]	Prospective	11	12 weeks	46 years (NA)	Octreotide LAR 30 mg/2-4 weeks (100 %)	458 ng/ml (67)	15 mg daily	195 ng/ml (24)	91	NA
Bianchi et al. [95]	Retrospective	27	30 weeks (6–72)	31 years (median age at diagnosis)	Lanreotide ATG 120 mg/4 weeks (63 %) Octreotide LAR 30 mg/4 weeks (37 %)	661 ng/ml (162)	20 mg daily (10–40)	372 ng/ml (216)	55.5	11.1

*SRLs* somatostatin receptor ligands, *PEG* Pegvisomant, *EOS* end of study, *TLEE* transient liver enzyme elevation

## General and tumor growth safety

### General safety

In clinical trials, Pegvisomant has been shown to be generally safe and well tolerated [29, 30] (HQ). In a global non-interventional surveillance study (1,288 subjects, mean duration 3.7 years) Pegvisomant-related adverse events (AE) (changes in tumor size, increase in liver enzymes, and injection site reactions) were recorded in 9.6 % of subjects [39]. In all studies, mortality was not related to Pegvisomant use (MQ). Injection-site reactions were initially reported with a frequency up to 11 % and were generally mild, erythematous, self-limited and did not require treatment [29, 30]. Lipodistrophy during Pegvisomant therapy was sporadically reported likely due to local lypolitic GH inhibition (LQ). Frequent rotation of injection sites could prevent local reactions and patients should be carefully monitored and trained [97, 98] (SR). Surveillance studies [33, 98] reported an elevation of liver transaminase levels > 3 times  ULN in about 5–8 % of patients mainly previously treated with SRLs. Transaminase level elevations during Pegvisomant treatment were often mild and transient, did not appear to be dose-related (idiosyncratic drug toxicity?) and occurred within the first year of treatment (MQ). Rare cases of drug-induced hepatitis (but not liver failure) were reported (VLQ) [99]. When Pegvisomant was combined with SRLs, transient liver enzyme elevations seemed to be 2–3 times more frequent (MQ) [33, 39, 82, 99–103]. Controversial is the correlation between diabetes mellitus and elevated transaminase levels (VLQ) [33, 92, 99, 101]. A common polymorphism found in Gilbert’s syndrome was associated with Pegvisomant-induced liver injury [104]. Biliary complications may arise from restitution to normal of gallbladder motility after cessation of SRL treatment [10]. We recommend not to start Pegvisomant if there is a liver dysfunction (SR). Liver function should be evaluated monthly for at least 6 months after initiating therapy, quarterly for next 6 months, and then semi-annually (SR). If transaminases increase >5 times ULN or >3 times ULN with increased serum bilirubin Pegvisomant must be discontinued (SR). If transaminases increase < 3 times ULN without signs or symptoms of liver failure Pegvisomant could be continued (DR), but they must be monitored weekly (SR) [24, 29, 33]. Since Pegvisomant may improve glucose tolerance, glucose levels should be monitored particularly in first months of treatment and anti-diabetic drugs adjusted if necessary (DR) [30, 33].

### Tumor growth safety

Only 1 out of 43 subjects treated with Pegvisomant for 29 months and monitored for 58 months, showed an increase in pituitary tumor volume [105]. In the German Pegvisomant Observational Study [106] in 18 out of 307 (5.9 %) patients treated with Pegvisomant for an average of 86 weeks tumor size increased; however, after centralized image re-evaluation, tumor progression was confirmed in only eight patients (3 %). Among 61 patients observed by Buhk et al. [107], in 3 (4.9 %) increased tumor volume >25 % during the first year of therapy was reported. Marazuela et al. [37] observed significant increased tumor size in 6.7 % of subjects (5 of 75), followed for 29 ± 20 months; absence of previous irradiation and shorter duration of pre-Pegvisomant SRL therapy were associated with increased risk of growth (LQ). In the global surveillance study [39] incidence of increased pituitary tumor size was 7.2 % (67 of 936) in the local MRI reading, while again it was only 3.2 % (45 of 936) in the central reading. Thus, a careful serial evaluation of all available images is necessary to avoid misinterpretations (SR) [39, 106]. Therefore, tumor growth, observed more frequently during the first year of treatment, may prevalently reflect the disease natural history [24, 30] or the consequence of SRL discontinuation [106]. On the contrary, irradiation seems to be associated with a reduction in tumor size [24, 105, 108]. All patients treated with Pegvisomant should undergo regular sellar MRI to screen for potential tumor growth (SR). A more intensive MRI follow-up protocol should be followed in non-irradiated patients (DR).

## Regulatory and cost/efficacy issues

### Regulatory issues

Pegvisomant was licensed for the treatment of acromegaly in 2002 by EMA (EU, European Medicines Agency) and in 2003 by FDA (US, Food and Drug Administration). Label indications in EU limit use of Pegvisomant to patients with acromegaly with inadequate response to surgery and/or radiation therapy and in whom medical treatment with SRLs did not normalize IGF-I or was not tolerated (third line therapy). Label indications in US indicate Pegvisomant in acromegaly patients with inadequate response to surgery and/or radiation therapy and/or other medical therapies, or for whom these therapies are not appropriate (first/second line therapy in specific cases) better reflecting available guidelines (MQ) [1–3]. Pegvisomant should be prescribed by doctors with expertise in acromegaly management (MQ). National and regional regulatory agencies provide largely variable criteria to allow centers for prescription (VLQ). First injection of Pegvisomant should be done under close medical supervision (SR) and specific warnings about systemic hypersensitivity reactions were recently added in the package leaflet. Injections less frequently than daily normalize IGF-I levels in some patients [108] and in Acrostudy [39] 12 % of clinicians did not use daily injections (VLQ). Combination therapy Pegvisomant + SRLs is not recommended by EMA though the Agency recognized the interest for the complementary actions of these drugs. Pegvisomant in combination therapy is considered an “off-label” use by some local regulatory agencies. Pegvisomant should not be used during pregnancy unless clearly necessary according to EMA and FDA (MQ) (pregnancy class B). In fact, there are only few reports about its safety in pregnancy [109].

### Cost/efficacy analysis

Pegvisomant is an effective but expensive drug (MQ). Certainly, the direct costs of neurosurgery, dopaminergic agents, SRLs and radiotherapy are lower than lifelong Pegvisomant treatment, but standard therapies do not provide biochemical normalization in some patients (HQ). On the other hand, control of disease is associated with normalized mortality rate and improvement of comorbidities (HQ) [1–3]. In addition, burden of direct and indirect (associated comorbidities and loss of working days) costs is higher in patients with acromegaly not controlled by standard therapies (MQ) [110, 111]. Therefore, if Pegvisomant is prescribed according to licensed use it may be cost-effective considering relative rarity of acromegaly (MQ). Nevertheless, according to a pharmacoeconomic model [112] the best cost-effectiveness ratio could be reached with Pegvisomant price reduced by about one-third (VLQ).

## Summary of recommendations

### Place of Pegvisomant in guidelines

#### Primary treatment

Pegvisomant cannot be recommended as primary treatment of the general acromegaly population (SR). In fact, surgery (performed by an experienced neurosurgeon) remains the primary treatment option in patients with acromegaly with totally resectable tumor (SR). Moreover, SRLs are primary medical treatment if surgery is contraindicated, not accepted by the patient or in case of poor likelihood of total surgical resection (SR). When surgery and radiation therapy cannot be performed and SRL are unlikely to be, or may not be, effective as in patients with acromegaly and McCune Albright syndrome or empty sella [113] Pegvisomant could be considered as primary treatment option (DR).

#### First-line (post-surgery) pharmacologic treatment

SRLs are primary first-line therapy after surgery (SR). Primary postsurgical therapy with cabergoline may be considered particularly in patients with relatively mild disease [114] (DR). There are at least three circumstances in which primary postsurgical medical treatment with Pegvisomant could be considered (DR): (1) patients who underwent a sufficiently long (>3–6 months) trial of pre-surgical SRL treatment [3] that was ineffective in controlling GH and IGF-1 and in whom mass effect of residual tumor is not an issue; (2) patients with residual tumor in whom radiation treatment is given as second option: in fact, after radiation elevated IGF-1 levels may persist for long time but likelihood of tumor regrowth is modest [1]; (3) patients with poorly controlled diabetes mellitus in whom SRL treatment may potentially worsen glucose metabolism [51–54].

#### Second-line pharmacologic treatment

**Fig. 1:**
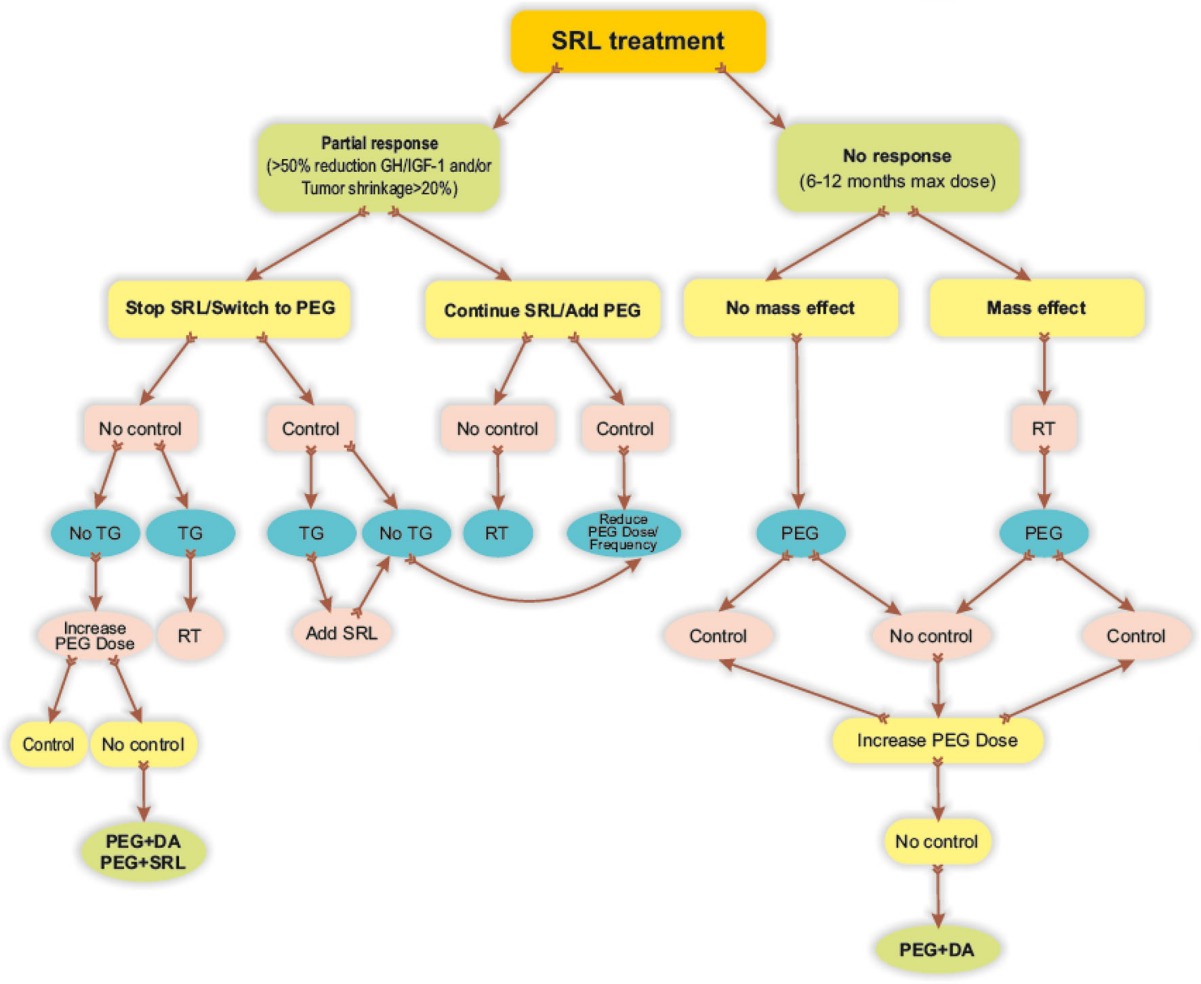
Proposed algorithm for the use of Pegvisomant in acromegaly patients partially or not responder to SRLs. *SRL* somatostatin receptor ligands, *PEG* Pegvisomant, *RT* radiation therapy, *TG* tumor growth, *DA* dopamine agonists

Partial (GH and IGF-I decreased but not normalized) or no response (minimal changes in GH and IGF-1) to SRLs may be observed (HQ) [3]. Patients with no response after an adequately long (6–12 months) period of treatment with maximal doses of SRL should be switched to Pegvisomant monotherapy (SR). If biochemical control is not achieved Pegvisomant dose should be increased (SR) and/or combination treatment with dopamine agonists should be given (DR). In patients who do not achieve biochemical control of the disease [7] but have documented partial response to SRLs (>50 % reduction of GH and IGF-1 vs. baseline and/or tumor shrinkage >20 %) either switching to Pegvisomant monotherapy or combination therapy Pegvisomant + SRL should be considered (DR). If SRL + Pegvisomant combination is not effective a possible alternative could be association of Pegvisomant with dopamine agonists (DR) [3] (Fig. 1). Patients seldom do not tolerate SRL treatment for gastrointestinal side effects (LQ) [115]: these subjects should be switched to Pegvisomant monotherapy regardless biochemical efficacy of SRL (taking into account potential mass effect) (SR).

### Dose, efficacy and safety monitoring

Individual optimal dose of Pegvisomant may vary according to anthropometric and genetic characteristics (VLQ). Recommended starting dose is 10 mg/day s.c. (DR). An initial load dose of Pegvisomant is not recommended (DR). Doses of Pegvisomant exceeding 30 mg/day are not recommended although in biochemically and clinically persistently active disease with no other treatment choice a further dose increase to 40 mg/day could be considered (DR).

Growth hormone should not be measured to assess effects of Pegvisomant (SR). Goal of Pegvisomant treatment is to normalize circulating IGF-1 levels (SR). Biochemical effects of Pegvisomant should be checked in laboratories with experience in IGF-1 measurement which give reference values divided by decade of age (SR). Patients with deranged glucose homeostasis on SRLs should be switched to Pegvisomant (DR). SRL treatment is known to counteract myocardial hypertrophy in patients with acromegaly [116] (HQ). Pegvisomant was also associated with positive cardiovascular effects and acromegaly cardiopathy does not contraindicate Pegvisomant (SR). Pegvisomant is the only treatment which was shown to normalize bone turnover in acromegaly [71] and prevalent vertebral fractures do not contraindicate Pegvisomant (DR).

Patients with known liver dysfunction should not be initiated with Pegvisomant (SR). Liver function should be evaluated periodically during therapy (SR). Injection-site reactions, such as lipodystrophy or lipohypertrophy may rarely occur and frequent rotation of injection sites is recommended (SR). Unlike SRLs [78–80] Pegvisomant treatment does not target tumor (HQ). Therefore, regular MRI monitoring is required (SR).
